# Supplementary figures and images for: Predicting Chemical Environments of Bacteria from Receptor Signaling
Source: PLoS Comput Biol. 2014 Oct 23;10(10):e1003870. doi: 10.1371/journal.pcbi.1003870 (PMC4207464; doi:10.1371/journal.pcbi.1003870)

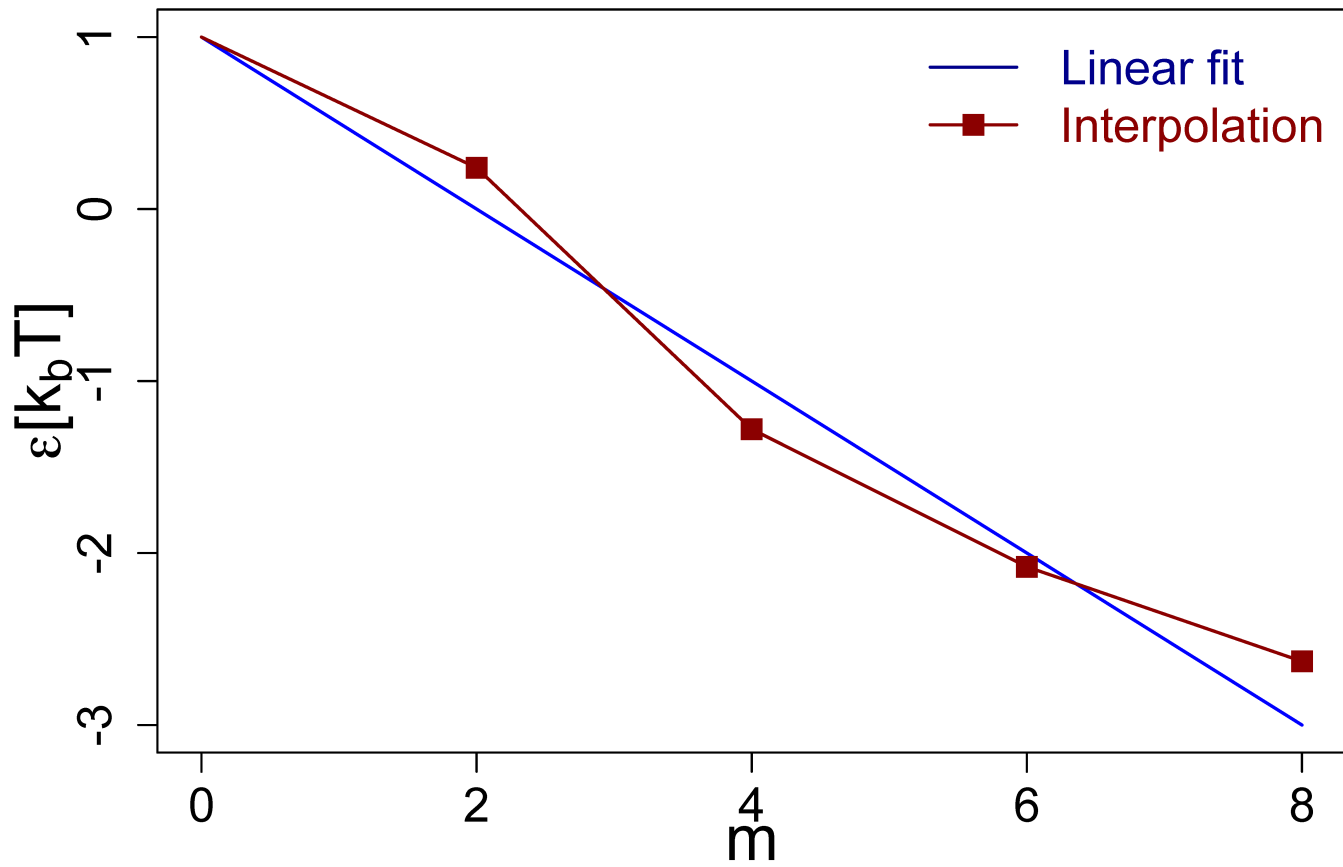

Supplement: Figure S2 — The offset energy as a function of modification level m . Comparison between linear fit [32] (blue) and interpolation of experimental data (red). (PDF) [file pcbi.1003870.s002.pdf]

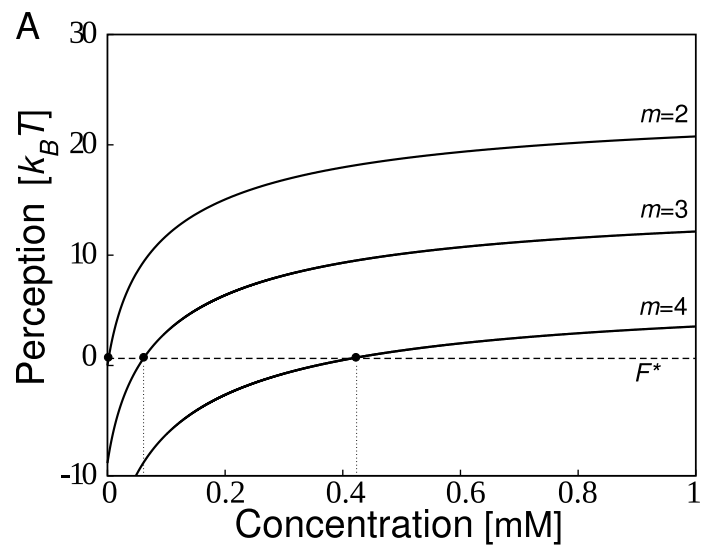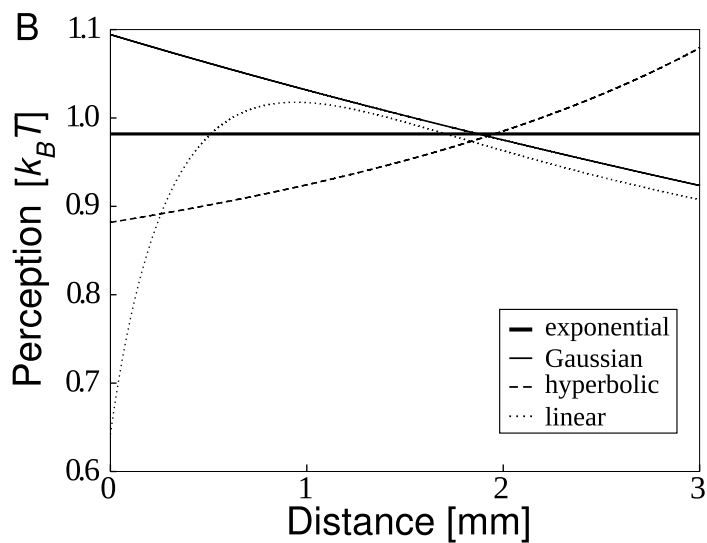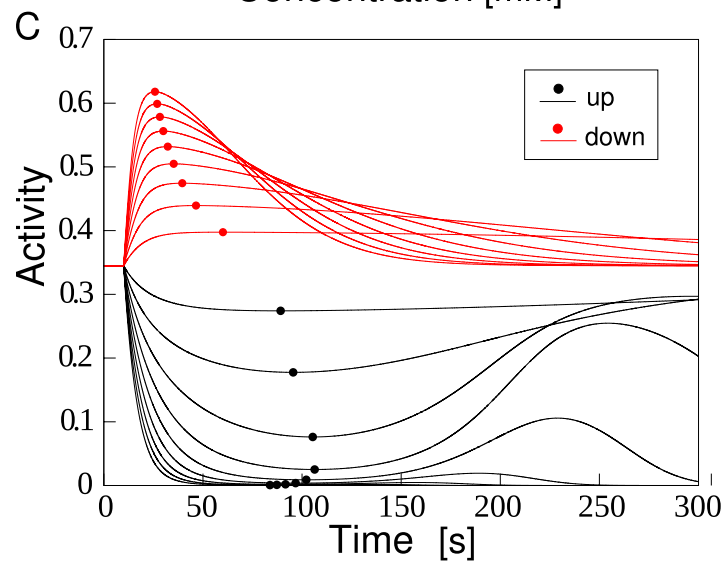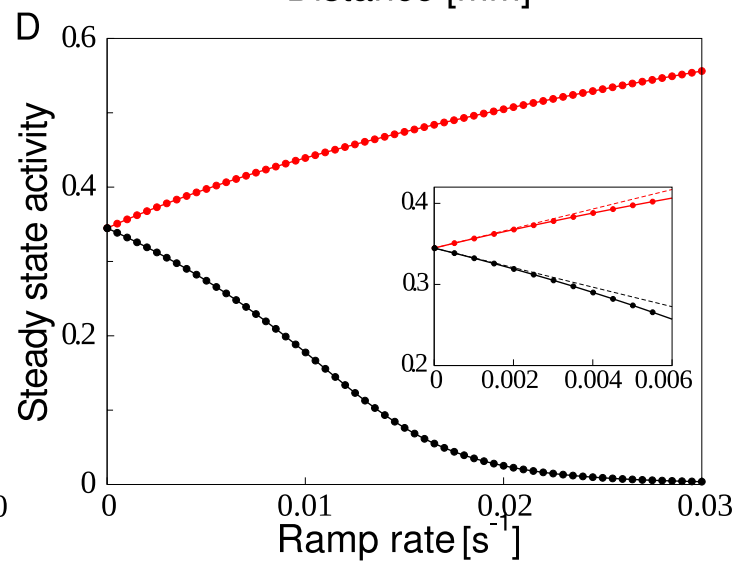

Supplement: Figure S3 — Perception in different chemical gradients. (A) Perception R of the Weber-Fechner law depending on ligand concentration c and modification level m. Adapted perception is given by the steady-state free-energy difference F * (dashed line). Three example curves corresponding to three different modification levels are plotted. On each curve, the adapted perception R = F * is indicated (solid circle) relating adapted modification level to the respective ambient concentration (dotted lines). (B) Perception along a straight swimming path in the respective concentration profile for swimming velocity vs = 20 µm s−1 and free-energy difference (in units of k B T). (C–D) Receptor complex activity for exponential concentration ramps, c(t) = c 0 e ±rt with ramp rate ±r. Results for up (+r, black) and down (−r, red) ramps for initial concentration c 0 = 0.1 mM are shown. (C) Time courses of receptor complex activity for ramps starting at t = 10 s with increasing rates r (low rates correspond to small changes in the receptor complex activity from the adapted state). Dots indicate the times when dA/dt = 0 (plateau) is reached for the first time. (D) Plateau activity as function of rate r. (B Inset) Same as B, but only for small rates. The dashed lines represent our analytical result. (PDF) [file pcbi.1003870.s003.pdf]

A

No rotational diffusion

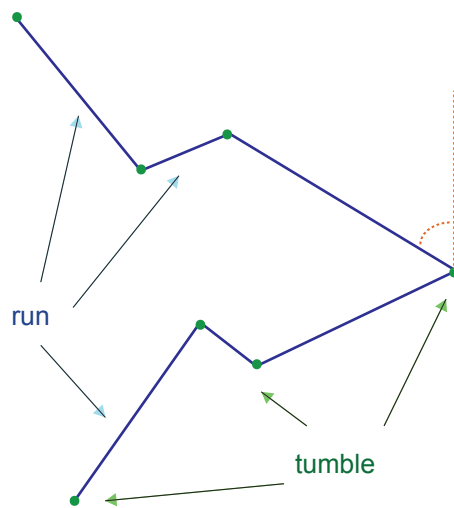

B

With rotational diffusion

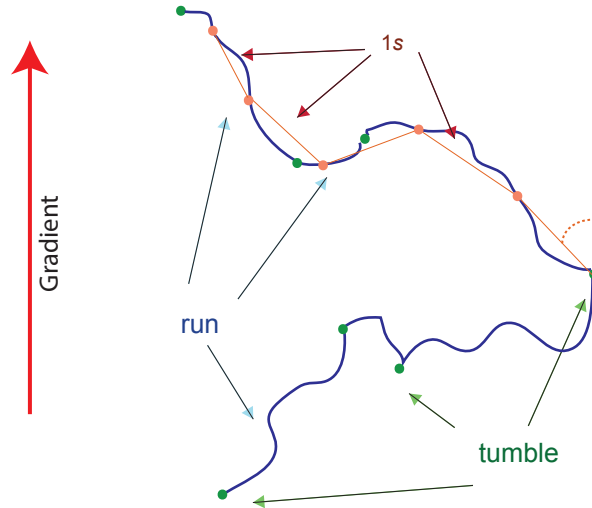

C

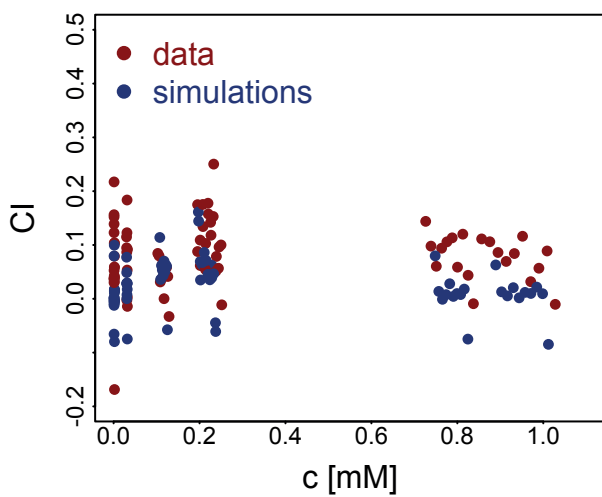

D

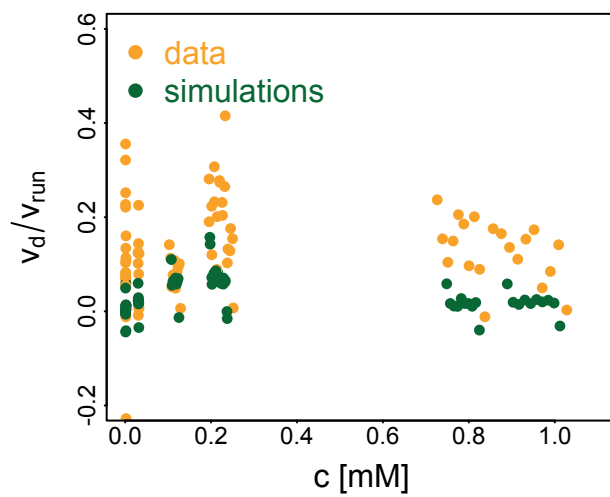

E

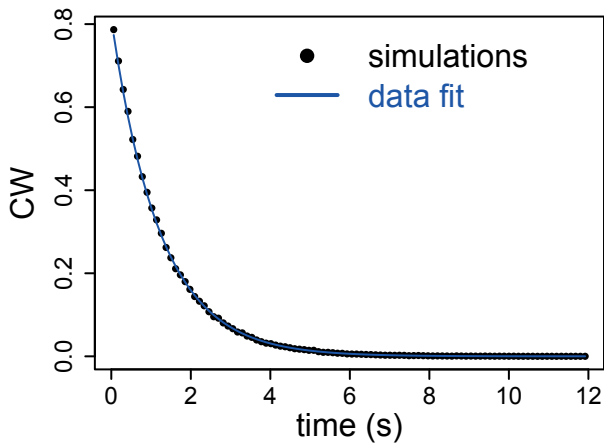

F

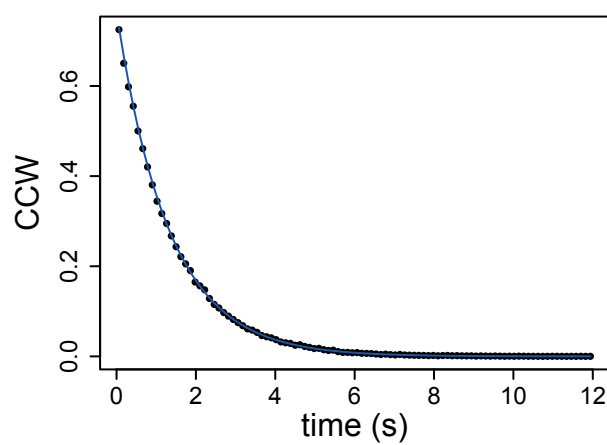

Supplement: Figure S5 — Calculation of the chemotactic index (CI) and comparison between simulations and data. (A–B) Schematics of trajectories of a swimming cell and calculation of CI with gradient in vertical direction. (A) Without rotational diffusion run length lk and angle θk between run direction and gradient are well defined between two tumbling events. (B) Rotational diffusion curves runs. To allow calculation of CI we use a linear-piecewise approximation of the trajectory using time step Δt = 0.1 s, allowing us to define lk and θk. (C–D) Average CI (C) and drift velocity (D) as a function of the concentration in which wild-type E. coli bacteria swim. Simulations (in blue and green, respectively) match tracking experiments from [50] (in red and orange, respectively) using identical shallow linear gradients. (E–F) Simulated clockwise (CW) rotation (E) and counter-clockwise (CCW) rotation (B) of single-motor interval distribution (black dots) of adapted cells match the exponential fits of experimental data [44] (blue lines). Decay time is close to (A), is close to (B). (PDF) [file pcbi.1003870.s005.pdf]

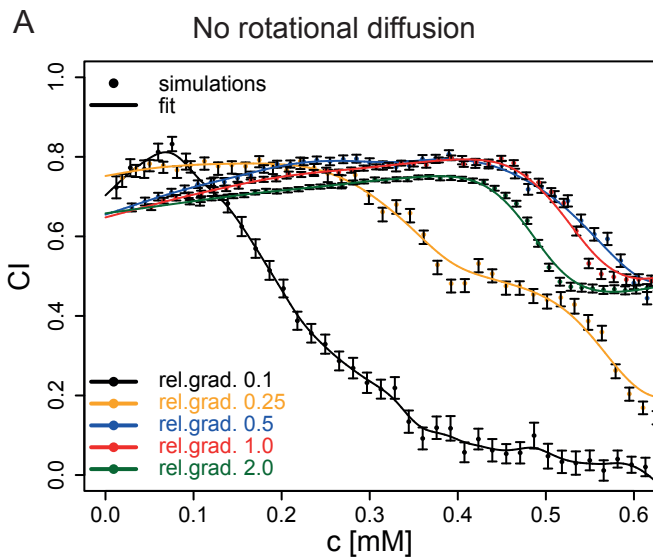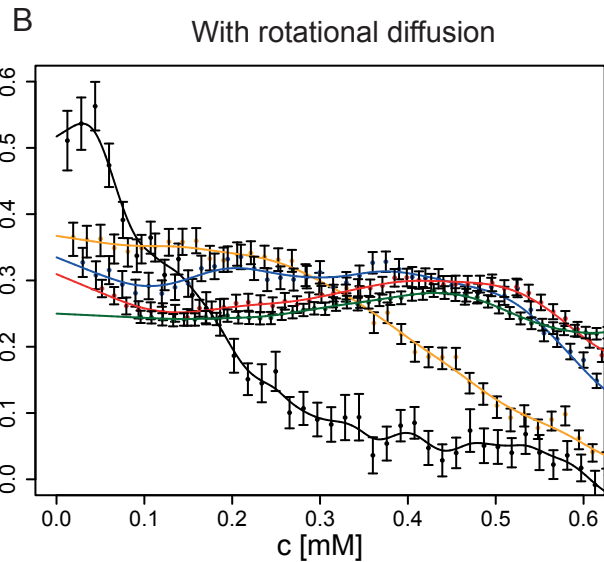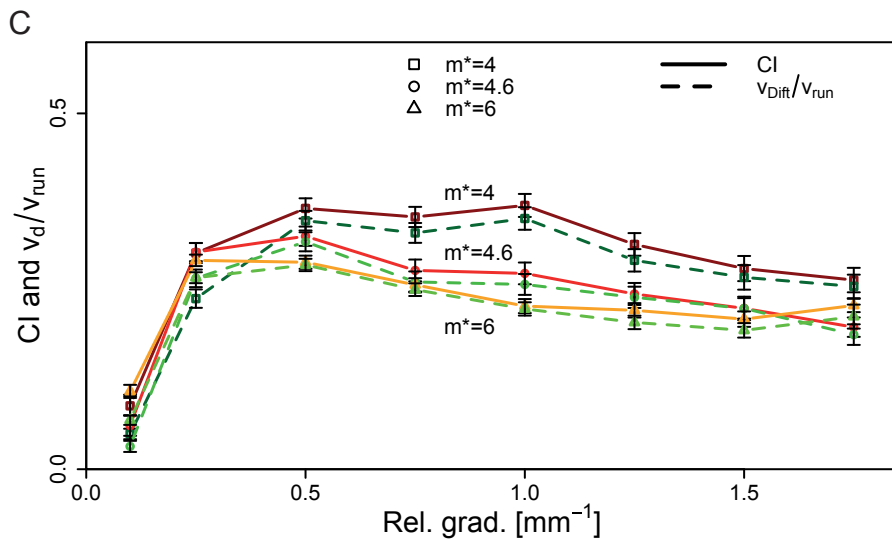

Supplement: Figure S6 — Additional results for chemotactic index (CI). (A–B) CI as a function of concentration in different linear gradients (in units of mm −1). (A) Simulations without rotational diffusion significantly increase CI compared to simulations with rotational diffusion (B). Although there is no clear maximum for each linear gradient with rotational diffusion, there is a linear gradient that maximizes CI for each concentration in both cases. Linear gradients relative to c * = 0.2239 mM with value 0.1 in black, 0.25 in yellow, 0.5 in blue, 1.0 in red and 2.0 in green in unit of mm −1. (C) Comparison of CI (red scale solid lines) and drift velocity (green scale dotted lines; relative to run velocity assumed to be constant with ). This graph shows the close similarity of the two quantities in line with Eq. 27 in Text S1. Symbols indicate different receptor-modification levels with m * = 4 corresponding to QEQE (squares), m * = 4.6 corresponding to WT 2 (0.1 mM) (circles), and m * = 6 corresponding to QEQQ (triangles). (PDF) [file pcbi.1003870.s006.pdf]

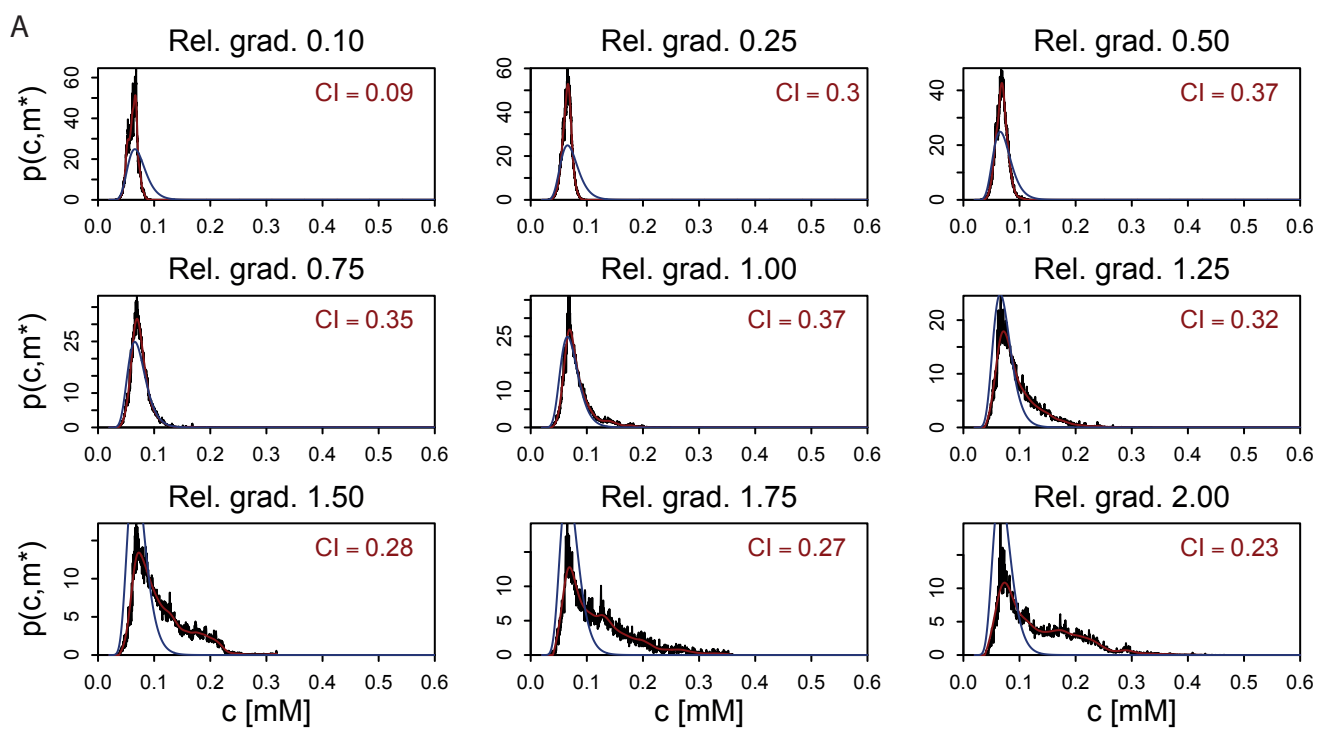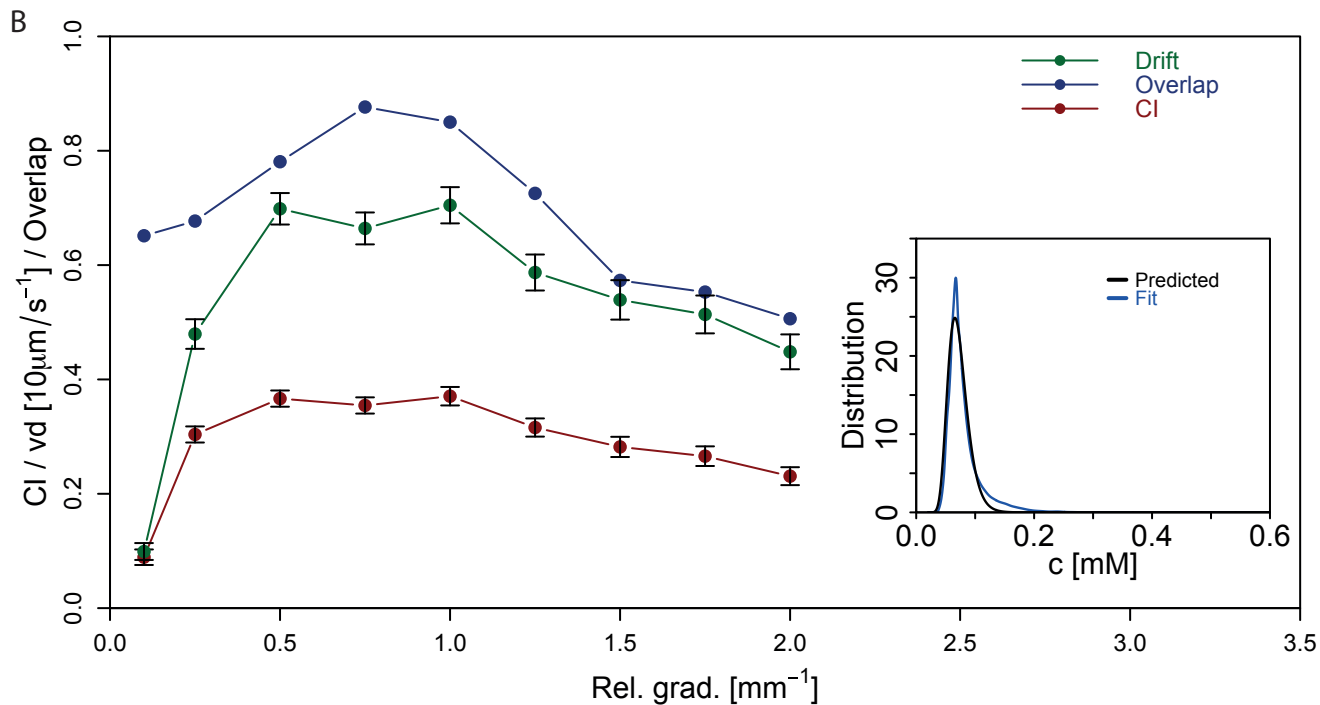

Supplement: Figure S7 — Comparison between simulated and predicted distribution of inputs for m * = 4 (QEQE). (A) Distributions of input concentration from simulations (red) and information theory predictions (blue) for different relative linear gradients. Corresponding chemotactic index (CI) is shown for each panel. Receptor-modification level is selected with Gaussian distribution with standard deviation 0.04% to mimic cell-internal (output) noise. The gradient shift up and down with Gaussian distribution with standard deviation 0.001% to mimic cell-external (input) noise. (B) Overlap between simulated and predicted distributions of inputs (dark blue), CI (red), and drift velocity (green) as a function of the relative gradient. (Inset) Fit to predicted distribution using simulated distribution of inputs in panel A as bases set. Overlap 89.4% between fit and prediction. (PDF) [file pcbi.1003870.s007.pdf]

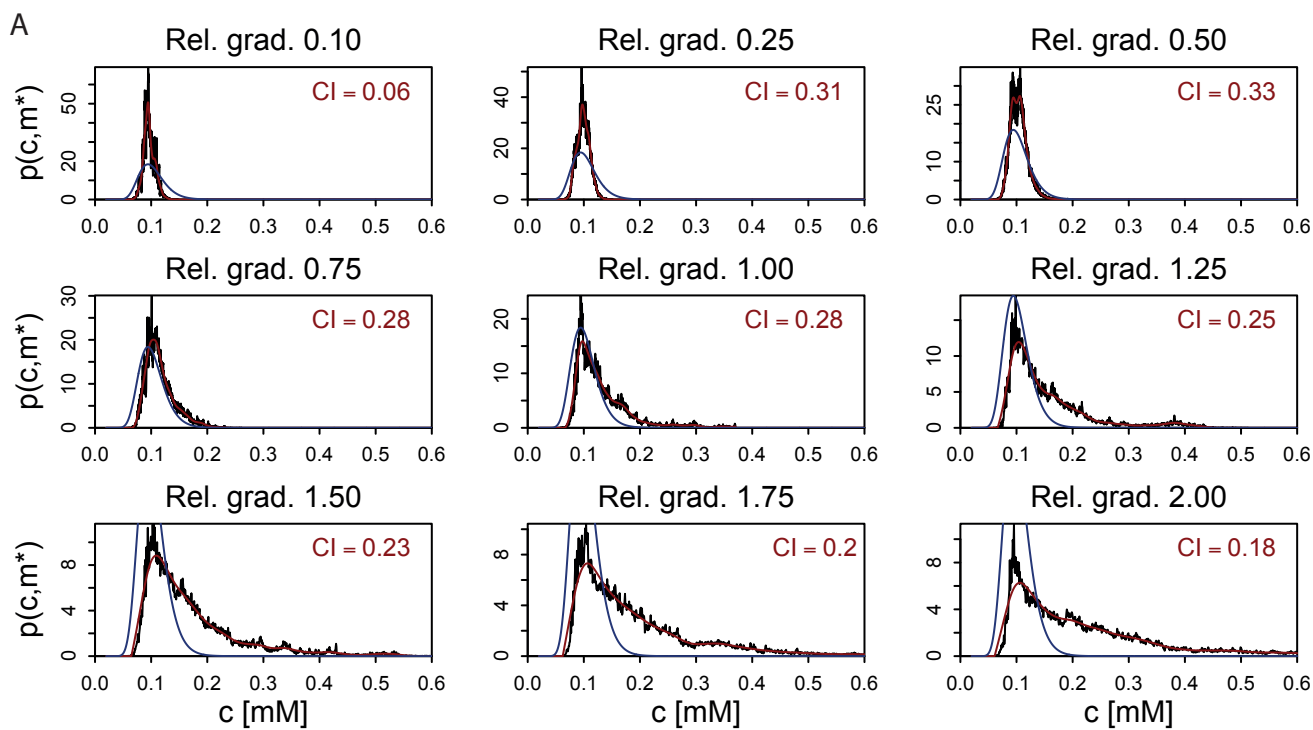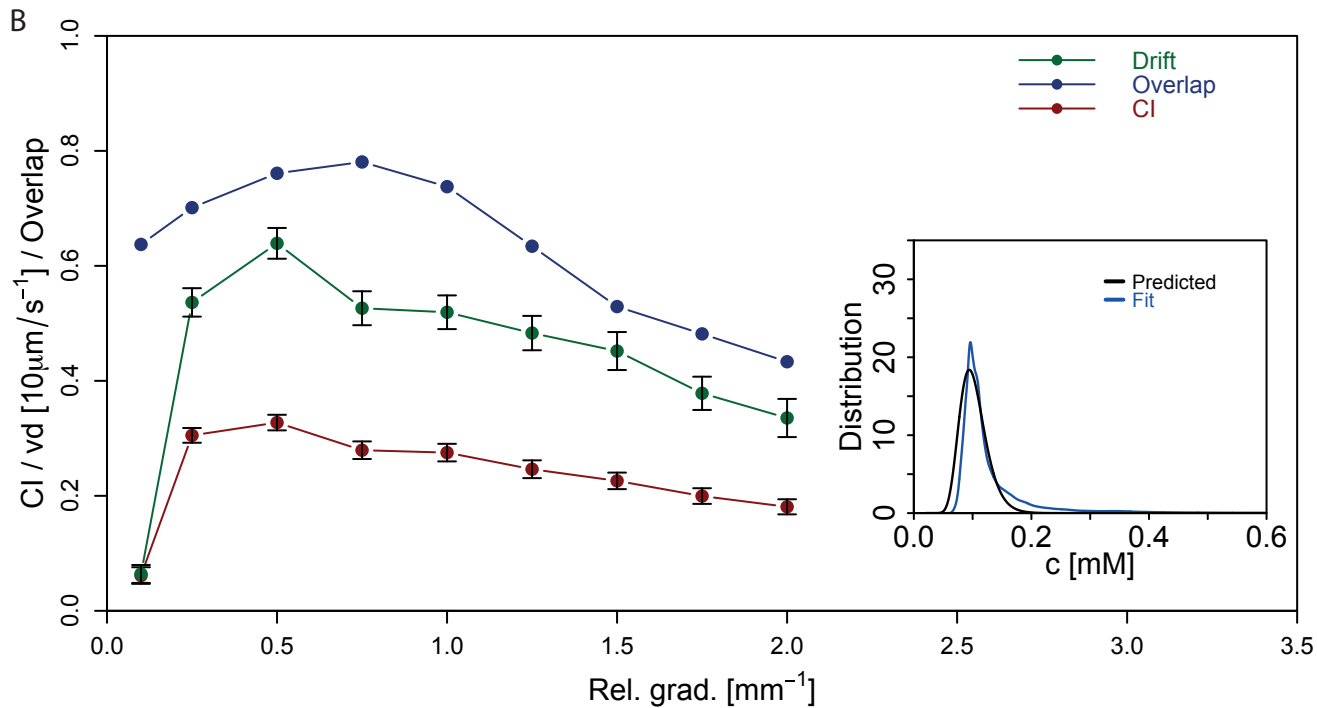

Supplement: Figure S8 — Comparison between simulated and predicted distribution of inputs for m * = 4.6 (WT 2 0.1 mM). (A) Distributions of input concentration from simulations (red) and information theory predictions (blue) for different relative linear gradients. Corresponding chemotactic index (CI) is shown for each panel. Receptor-modification level is selected with Gaussian distribution with standard deviation 0.04% to mimic cell-internal (output) noise. The gradient shift up and down with Gaussian distribution with standard deviation 0.001% to mimic cell-external (input) noise. (B) Overlap between simulated and predicted distributions of inputs (dark blue), CI (red), and drift velocity (green) as a function of the relative gradient. (Inset) Fit to predicted distribution using simulated distribution of inputs in panel A as bases set. Overlap 79.4% between fit and prediction. (PDF) [file pcbi.1003870.s008.pdf]

A

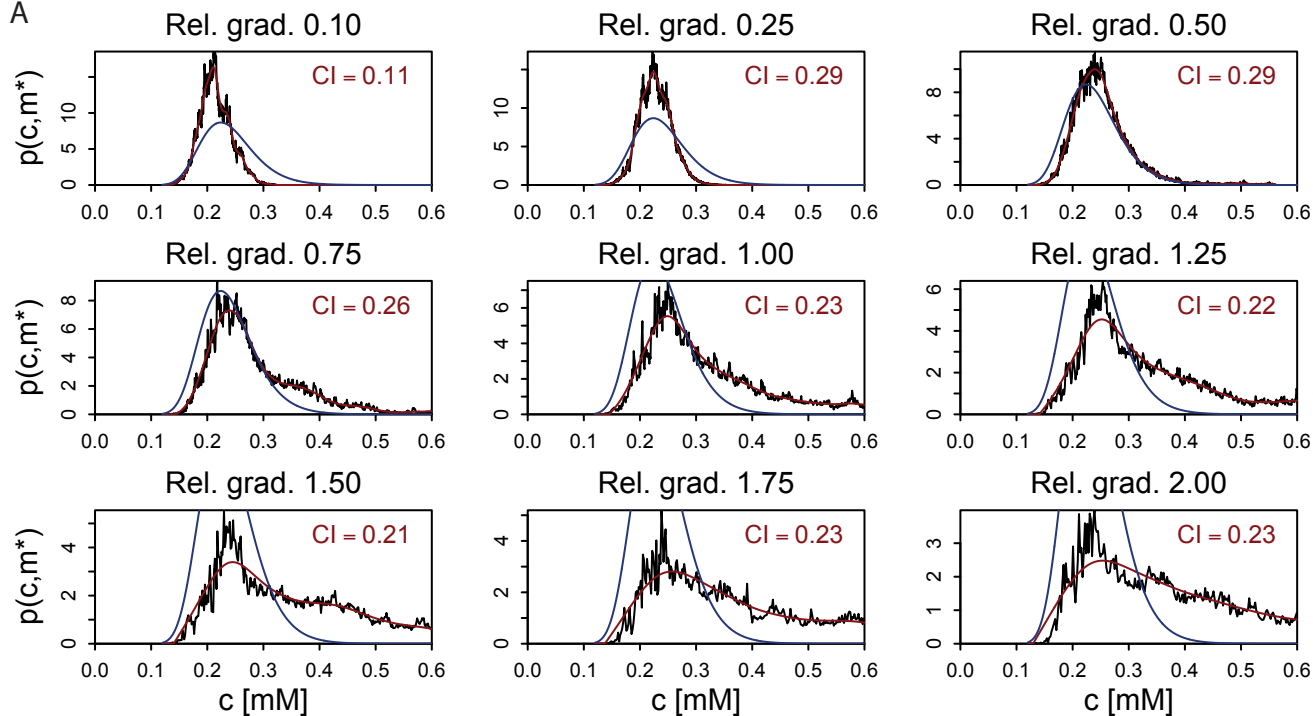

B

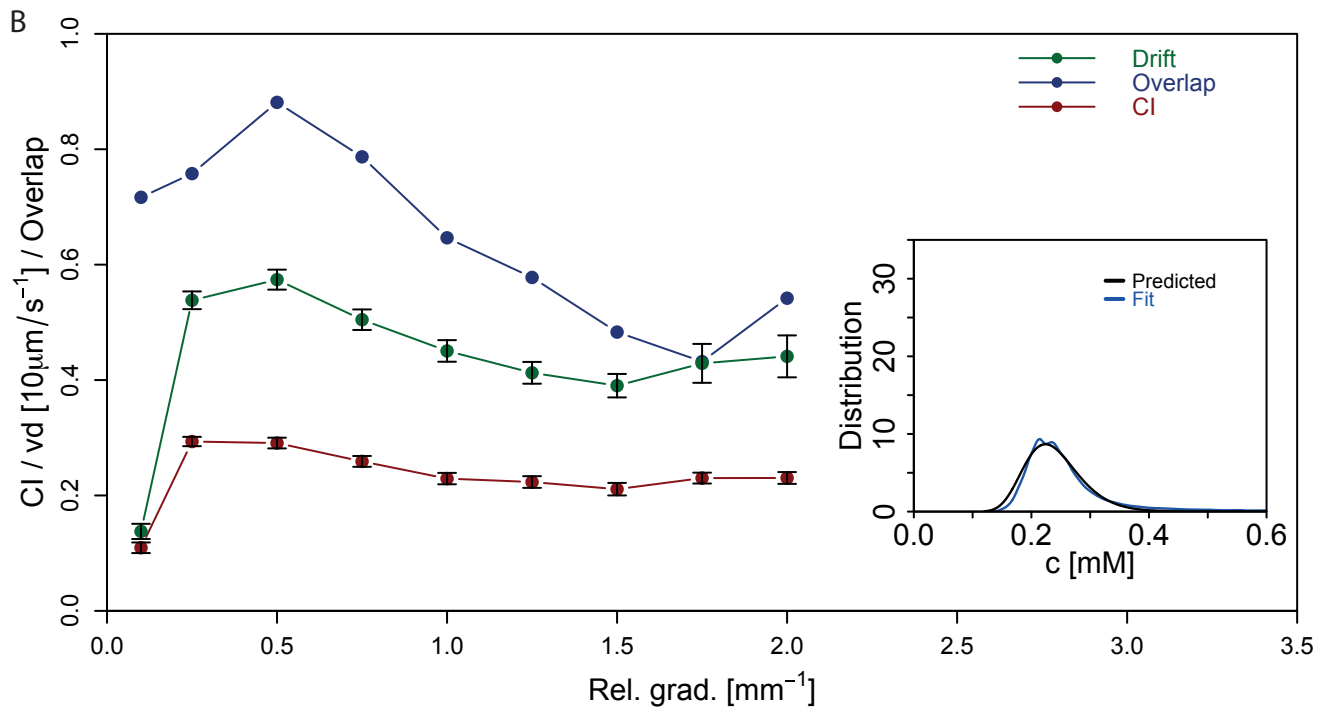

Supplement: Figure S9 — Comparison between simulated and predicted distribution of inputs for m * = 6 (QEQQ). (A) Distributions of input concentration from simulations (red) and information theory predictions (blue) for different relative linear gradients. Corresponding chemotactic index (CI) is shown for each panel. Receptor-modification level is selected with Gaussian distribution with standard deviation 0.04% to mimic cell-internal (output) noise. The gradient shift up and down with Gaussian distribution with standard deviation 0.001% to mimic cell-external (input) noise. (B) Overlap between simulated and predicted distributions of inputs (dark blue), CI (red), and drift velocity (green) as a function of the relative gradient. (Inset) Fit to predicted distribution using simulated distribution of inputs in panel A as bases set. Overlap 91.5% between fit and prediction. (PDF) [file pcbi.1003870.s009.pdf]

A

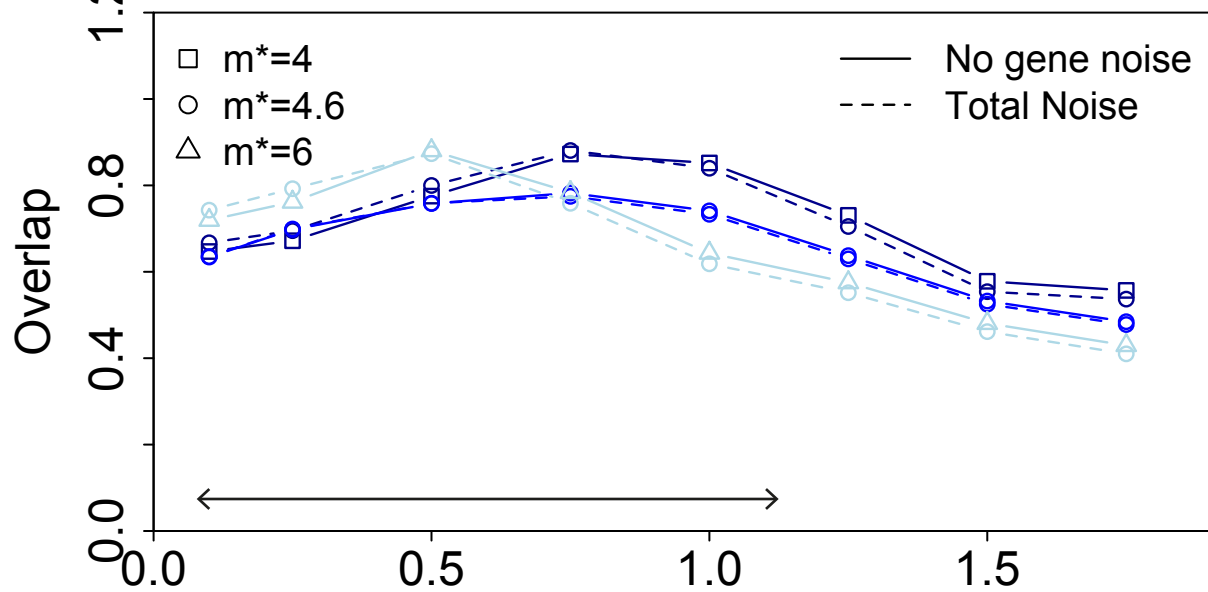

B

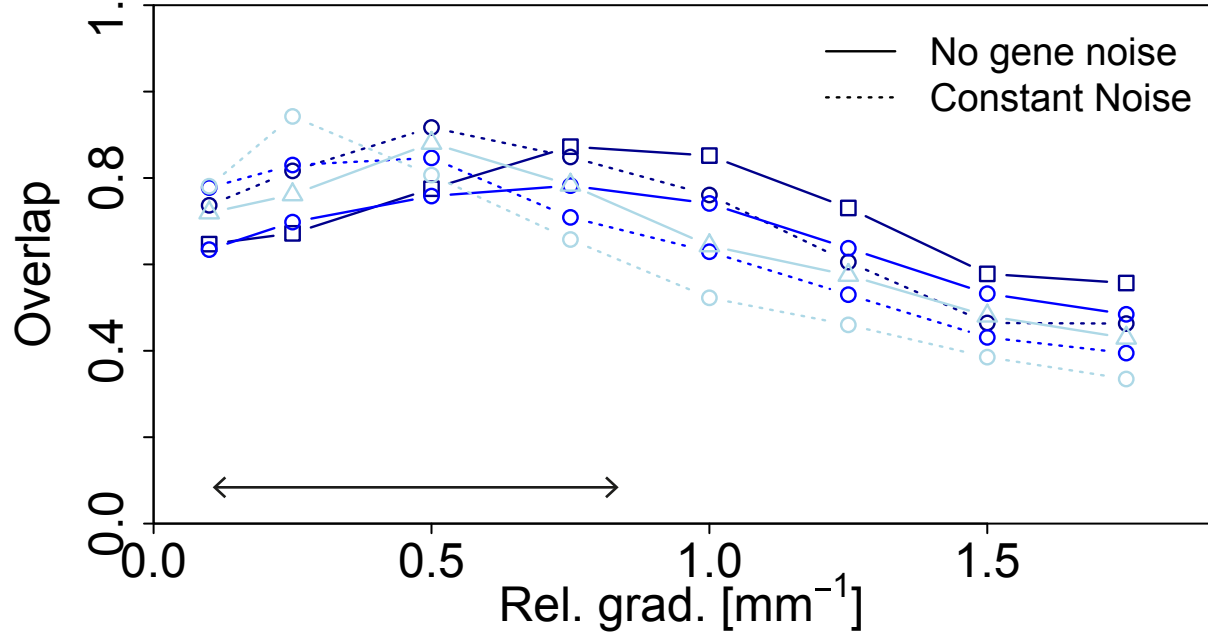

Supplement: Figure S10 — Distribution of relative gradients from overlap between distributions of sampled concentrations and predicted distributions. (A) Predicted distributions excluding gene-expression noise (all principal components except the first; solid lines) and distributions predicted with total noise including gene-expression noise (all principal components; dashed lines). Results look very similar showing robustness of our predictions from information theory and simulations. (B) Predicted distributions excluding gene-expression noise (all principal components except the first; solid lines) and distributions predicted with uniform (constant) output noise (dotted lines). Maximum overlap in the latter case shifts to shallower gradients since predicted input distributions are narrower and more symmetric (cf. Fig. S4). Horizontal arrow illustrates range of relative gradients over which the overlap is within 20% of maximal value on average for total noise (A) and uniform noise (B), cf. Fig. 5B in the main text. (PDF) [file pcbi.1003870.s010.pdf]
