# Supplementary material for: Predicting Chemical Environments of Bacteria from Receptor Signaling
Source: PLoS Comput Biol. 2014 Oct 23;10(10):e1003870. doi: 10.1371/journal.pcbi.1003870 (PMC4207464; doi:10.1371/journal.pcbi.1003870)
Supplement: Table S1 — Fit parameters of the variance in FRET activity. Parameter values for the noise components of FRET activity (cf. Eq. 11 in the main text) excluding (top) and including (bottom) gene-expression noise, respectively. Corresponding 95% confidence intervals calculated using the profile-likelihood approach are given in brackets below the fitted value. We set α 1 and α 2 to zero when below 10−20. The goodness-of-fit is indicated by the value of each fit in comparison to the critical value corresponding to a significance level of 0.05 (given in brackets below the value). If , the model is consistent with the data and classified as a good fit (last column). (PDF) [file pcbi.1003870.s011.pdf]

Strain	α_1 (confidence interval)	α_2 (confidence interval)	α_3 (confidence interval)	χ^2 (χ^2_{crit})	good fit
**Excluding gene-expression noise**					
WT 2 (0 mM)	NA	NA	$1.158 \cdot 10^{-8}$ $(0, 2.584 \cdot 10^{-8})$	6.08 (11.07)	yes
WT 2 (0.1 mM)	$3.910 \cdot 10^{-14}$ $(0, 2.398 \cdot 10^{-13})$	$3.344 \cdot 10^{-8}$ $(1.927 \cdot 10^{-8}, 4.731 \cdot 10^{-8})$	$2.165 \cdot 10^{-9}$ $(0, 5.989 \cdot 10^{-9})$	13.24 (18.31)	yes
QEEE	$2.428 \cdot 10^{-13}$ $(0, 1.804 \cdot 10^{-12})$	NA	$8.244 \cdot 10^{-9}$ $(4.701 \cdot 10^{-9}, 1.173 \cdot 10^{-8})$	6.35 (9.49)	yes
QEQE	$8.197 \cdot 10^{-15}$ $(0, 4.521 \cdot 10^{-13})$	$1.565 \cdot 10^{-7}$ $(1.200 \cdot 10^{-7}, 1.928 \cdot 10^{-7})$	$3.923 \cdot 10^{-9}$ $(0, 8.051 \cdot 10^{-9})$	6.85 (7.81)	yes
QEQQ	$3.494 \cdot 10^{-12}$ $(0, 9.358 \cdot 10^{-12})$	$1.298 \cdot 10^{-6}$ $(1.061 \cdot 10^{-6}, 1.521 \cdot 10^{-6})$	$1.400 \cdot 10^{-8}$ $(0, 4.910 \cdot 10^{-8})$	11.30 (14.07)	yes
QQQQ	0 $(0, 1.074 \cdot 10^{-11})$	$1.528 \cdot 10^{-7}$ $(0, 3.208 \cdot 10^{-7})$	$8.241 \cdot 10^{-8}$ $(5.620 \cdot 10^{-8}, 1.081 \cdot 10^{-7})$	10.56 (14.07)	yes
**Including gene-expression noise**					
WT 2 (0 mM)	NA	NA	$6.494 \cdot 10^{-8}$ $(3.656 \cdot 10^{-8}, 9.284 \cdot 10^{-8})$	6.02 (11.07)	yes
WT 2 (0.1 mM)	0 $(0, 2.273 \cdot 10^{-12})$	0 $(0, 1.384 \cdot 10^{-7})$	$1.525 \cdot 10^{-6}$ $(1.349 \cdot 10^{-6}, 1.702 \cdot 10^{-6})$	15.82 (18.31)	yes
QEEE	0 $(0, 2.297 \cdot 10^{-11})$	NA	$1.674 \cdot 10^{-6}$ $(1.553 \cdot 10^{-6}, 1.795 \cdot 10^{-6})$	6.80 (9.49)	yes
QEQE	0 $(0, 2.492 \cdot 10^{-12})$	$5.343 \cdot 10^{-7}$ $(2.932 \cdot 10^{-7}, 7.649 \cdot 10^{-7})$	$3.670 \cdot 10^{-7}$ $(3.403 \cdot 10^{-7}, 3.934 \cdot 10^{-7})$	6.02 (7.81)	yes
QEQQ	$2.459 \cdot 10^{-12}$ $(0, 6.727 \cdot 10^{-12})$	$1.200 \cdot 10^{-6}$ $(1.024 \cdot 10^{-6}, 1.376 \cdot 10^{-6})$	$2.014 \cdot 10^{-7}$ $(1.754 \cdot 10^{-7}, 2.274 \cdot 10^{-7})$	11.33 (14.07)	yes
QQQQ	$3.287 \cdot 10^{-11}$ $(0, 7.961 \cdot 10^{-11})$	$3.018 \cdot 10^{-6}$ $(2.662 \cdot 10^{-6}, 3.374 \cdot 10^{-6})$	$3.292 \cdot 10^{-7}$ $(2.742 \cdot 10^{-7}, 3.845 \cdot 10^{-7})$	11.90 (14.07)	yes
