# Supplementary material for: Predicting Chemical Environments of Bacteria from Receptor Signaling
Source: PLoS Comput Biol. 2014 Oct 23;10(10):e1003870. doi: 10.1371/journal.pcbi.1003870 (PMC4207464; doi:10.1371/journal.pcbi.1003870)
Supplement: Table S2 — Log-normal fits to predicted input distributions. Estimated parameter values for the fit of log-normal distributions (cf. Eq. 24 in Text S1) to the predicted input distributions shown in Fig. 3 in the main text and Fig. S4. Corresponding 95% confidence intervals are given in brackets below the fitted value. (PDF) [file pcbi.1003870.s012.pdf]

Strain	μ	σ	$\langle x \rangle$	$\frac{\langle x^2 - \langle x \rangle^2 \rangle}{\langle x \rangle^2}$
Excluding the first principal component				
WT 2 (0.1 mM)	-2.3975	0.2479	0.0938	0.0634
	(-2.3985, -2.3964)	(0.2471, 0.2488)		
QEQE	-2.7727	0.2695	0.0648	0.0753
	(-2.7736, -2.7717)	(0.2687, 0.2703)		
QEQQ	-1.5675	0.2242	0.2139	0.0515
	(-1.5684, -1.5666)	(0.2234, 0.2249)		
QQQQ	-0.4678	0.2492	0.6461	0.0641
	(-0.4696, -0.4660)	(0.2477, 0.2507)		
Excluding the first principal component				
WT 2 (0.1 mM)	-2.3222	0.2283	0.1006	0.0535
	(-2.3234, -2.3209)	(0.2272, 0.2293)		
QEQE	-2.7136	0.2387	0.0682	0.0586
	(-2.7148, -2.7124)	(0.2377, 0.2396)		
QEQQ	-1.5350	0.2022	0.2199	0.0417
	(-1.5361, -1.5338)	(0.2012, 0.2031)		
QQQQ	-0.5037	0.2560	0.6244	0.0677
	(-0.5053, -0.5020)	(0.2546, 0.2574)		
Constant output noise				
WT 2 (0.1 mM)	-2.4340	0.1811	0.0891	0.0333
	(-2.4346, -2.4333)	(0.1805, 0.1816)		
QEQE	-2.7984	0.1882	0.0620	0.0361
	(-2.7991, -2.7977)	(0.1877, 0.1888)		
QEQQ	-1.5790	0.1523	0.2086	0.0235
	(-1.5795, -1.5785)	(0.1519, 0.1527)		
QQQQ	-0.5492	0.1921	0.5882	0.0376
	(-0.5499, -0.5486)	(0.1916, 0.1926)		
