# Supplementary material for: Predicting Chemical Environments of Bacteria from Receptor Signaling
Source: PLoS Comput Biol. 2014 Oct 23;10(10):e1003870. doi: 10.1371/journal.pcbi.1003870 (PMC4207464; doi:10.1371/journal.pcbi.1003870)
Supplement: Text S1 — Model and simulation details and additional results. (PDF) [file pcbi.1003870.s013.pdf]

Supporting Text S1: Predicting chemical environments of bacteria from receptor signaling

Diana Clausznitzer[1,2,3,4,†], Gabriele Micali[1,2,†], Silke Neumann[5], Victor Sourjik[5,6] and Robert G. Endres[1,2,*]
1 Department of Life Sciences, Imperial College, SW7 2AZ London, UK
2 Centre for Integrative Systems Biology and Bioinformatics at Imperial College, SW7 2AZ, London, UK
3 BioQuant, Heidelberg University, 69120 Heidelberg, Germany
4 Institute for Medical Informatics and Biometry, Technische Universität Dresden, 01307 Dresden, Germany
5 Centre of Molecular Biology, Heidelberg University, DKFZ-ZMBH Alliance, 69120 Heidelberg, Germany
6 Max Planck Institute for Terrestrial Microbiology, D-35043 Marburg, Germany
† Both authors contributed equally to this work
∗ E-mail: r.endres@imperial.ac.uk

Introduction to fold-change detection and Weber's law

Many sensory systems display fold-change detection (FCD), i.e. show a response profile which depends on fold-changes, rather than absolute levels of an input stimulus (Fig. S1A, top panels). For instance, the human eye copes with 8 orders of magnitude of brightness from low light during a moonless night to bright daylight [1, 2]. To distinguish objects from their background the visual system efficiently exploits the statistical similarity of light intensities in natural scenes, with the relative difference between light reflected from the object compared to the background being largely invariant under different illuminations [1,3,4].

A special case of FCD is Weber's law. Historically, it was interpreted as describing the relationship between the physical magnitude of a stimulus and its perceived intensity [5]. Specifically, the law states that the smallest noticeable difference ΔS (threshold stimulus), which a sensory system can detect between a stimulus and a persistent background stimulus S_0, increases directly proportional to S_0 (Fig. S1A, bottom left panel). The ratio $\Delta S/S_0$ remains therefore constant, representing coding of contrast, i.e. stimuli relative to the background. Hence, Weber's law represents FCD in the linear response regime.

In its integrated form, Weber's law predicts an internal representation of the stimulus, the perception $R \sim \ln S$, to increase logarithmically with the stimulus S (Fig. S1A, bottom right panel). This relationship, known as the Weber-Fechner law [5], results in a logarithmic stimulus compression and hence a large dynamic range, found, e.g., in the visual and auditory system [2] and in the neural representation of numbers [6].

While such phenomenological laws apply to many sensory systems, an explanation at the molecular level is generally difficult due to the complexity of the underlying molecular and neural processes. In higher organisms, Weber's law is best documented in the insect and vertebrate visual system, where multiple adaptation mechanisms adjust the receptor sensitivity to ambient light levels [7]. In the insect compound eye, receptor cells remain sensitive to light over a wide range of intensities due to adaptation, as voltage-dependent potassium channels repolarize the cell and restrict the response amplitude at higher light intensities for stimulus compression [4]. In addition, further optical, cellular and neural adaptation mechanisms have been described, among them pigment migration [4], reduction of response latency in the transduction process [8], and transient activation of synaptic transmitter [9].

Introduction to information theory

Sensory systems adjust their signaling properties to match the typical input distributions they encounter. Appropriate matching of distributions of relevant inputs, input-output relationships and distributions of outputs has been found in many biological systems [10–14]. In the simplest case with constant output noise, Laughlin suggested that inputs should be encoded into outputs such that all available output levels are used equally often [10]. Having one input and one output variable, the mutual information is optimized if the input-output relationship is given by the cumulative distribution of the input, i.e. $\bar{A}(c) = \int^{I} dc' p_I(I')$, as illustrated in Fig. S1B.

Information transmission can be quantified using Shannon's mutual information [11–13, 15, 16]. For one input variable c and one output variable A the mutual information is given by

$$\begin{aligned}\mathcal{I}[c;A] &= \iint dc\, dA\ p(c,A) \log_2 \frac{p(c,A)}{p_c(c)\, p_A(A)} \\ &= \int dc\, p_c(c) \int dA\, p(A|c) \log_2 \frac{p(A|c)}{p_A(A)},\end{aligned} \tag{1}$$

where the joint probability $p(c, A)$ can be expressed in terms of the conditional probability $p(A|c)$ according to $p(c, A) = p(A|c)p_c(c)$.

We assume that for a given input value c the output is distributed normally around a mean output value $\bar{A}(c)$ with variance $\sigma_T^2(c)$ due to transmitted cell-external (input) noise and cell-internal (output) noise. Hence, the input-output relationship is given by the conditional probability for the output A given an input concentration c,

$$p(A|c) = \frac{1}{\sqrt{2\pi\sigma_T^2(c)}} \exp\left\{-\frac{[A - \bar{A}(c)]^2}{2\sigma_T^2(c)}\right\}. \tag{2}$$

Assuming small noise σ_T^2, the mutual information Eq. 1 reduces to Eq. 8 in the main text. Maximizing the mutual information according to the derivation given in *Materials and Methods* in the main text yields the relationship between dose-response curve and expected input distribution given by Eq. 9 in the main text.

In the absence of input noise, the optimal dose-response curve becomes the steeper the higher the variance of the output according to [13]

$$\frac{\partial \bar{A}}{\partial c} \propto \sigma_A \cdot p_c(c), \tag{3}$$

where σ_A is the output noise of A, hence the derivative of the optimal dose-response curve is proportional to the variance from internal signaling noise. Intuitively, the steeper the dose-response curve, the larger the separation between different output states becomes, turning into an advantage at high levels of output noise. In contrast, if input noise is present it is transmitted through the input-output relationship. Hence, increasing the steepness of the dose-response curve does not help information transmission as input noise is equally amplified similar to a deterministic input signal. Hence, the optimal dose-response curve is then given by the balance of transmitted input and output noise according to Eq. 9 in the main text.

Derivation of noise threshold in FRET activity

The activity threshold ΔA (cf. Fig. 2B of the main text) defines the minimum activity response to a stimulus in order for the cell to distinguish the stimulus reliably from the background noise of the activity. For convenience, we picked values of $\Delta A/A^* = 0.08$ and 0.16, in Fig. 2E in the main text. These

threshold values are in the linear regime of the dose-response curves. Here, we theoretically estimate the magnitude of the activity threshold due to fluctuations in ligand concentration, receptor methylation level and receptor-complex switching. We use a result for the power spectrum of fluctuations obtained in [17], and the fact that the variance is the integral over all frequencies of the power spectrum divided by 2π.

The power spectrum $S_A(\omega)$ of fluctuations in the total activity of N_C receptor complexes in a cell each containing N receptors is [17]

$$S_A(\omega) = N_C \frac{\omega^2 \left[S_a(\omega) + \left(\frac{\partial A}{\partial c}\right)^2 S_c(\omega) \right] + \left(\frac{\partial A}{\partial M}\right)^2 Q_M}{\omega_M^2 + \omega^2}, \tag{4}$$

where the individual terms in the numerator are the power spectrum for (*i*) receptor complex switching, $S_a(\omega)$, (*ii*) ligand noise, $S_c(\omega)$, and (*iii*) receptor-complex methylation Q_M. We assume that fluctuations at different receptor complexes are independent, hence all fluctuations are summed up yielding the factor N_C above.

According to Ref. [17], the individual contributions to the variance in activity from receptor-complex switching $\langle \delta A^2 \rangle_a$, receptor methylation $\langle \delta A^2 \rangle_M$, and ligand noise $\langle \delta A^2 \rangle_c$ are:

$$\langle \delta A^2 \rangle_a = \frac{2 N N_C A^* (1 - A^*)^2}{\pi k_2 \tau} \tag{5}$$

with k_2 the rate of *on*/*off* switching of a receptor complex and τ an averaging time due to slower downstream reactions,

$$\langle \delta A^2 \rangle_M = \frac{N^2 N_C (1 - A^*)^2 (A^*)^3}{3 - 2A^*}, \tag{6}$$

and

$$\langle \delta A^2 \rangle_c = N^2 N_C \left(\frac{\partial A}{\partial c} \right)^2 \langle \delta c^2 \rangle \tag{7}$$

with $\langle \delta c^2 \rangle = \alpha c_0 / (\pi a D \tau)$ [18, 19], where the parameters of the variance in ligand concentration denote a the size of the ligand binding site of a receptor (i.e receptor complex in our case), and D the ligand diffusion coefficient. Parameter α is of order one and depends on further receptor details. It is reduced for cooperative ligand binding by receptors [20] and is increased when ligand, once receptor-bound, is allowed to rebind to the same or a different receptor [19, 21]. The latter effect is enhanced by the close proximity of receptors in cell-polar clusters.

We assumed the following parameter values: adapted receptor activity $A^* = 1/3$, number of receptors per receptor-complex $N = 17.8$, number of receptor complexes $N_C = 449$ (corresponding to 8000 receptors per cell), a typical ligand concentration $c_0 = \sqrt{K_a^{\text{off}} K_a^{\text{on}}} = 0.1$ mM [22], receptor-switching rate $k_2 = 10^3$ s^{-1}, averaging time $\tau = k_A^{-1} = 0.1$ s corresponding to slow autophosphorylation of CheA, factor $\alpha \approx 1$, dimension of a receptor complex a=10 nm and diffusion coefficient D=100 μm^2/s. Therefore the contributions of the variance above evaluate to: $\langle \delta A^2 \rangle_a = 1.06 \cdot 10^{-6}$, $\langle \delta A^2 \rangle_M = 1.41 \cdot 10^{-4}$ and $\langle \delta A^2 \rangle_c = 0.0025$, respectively (in units of the adapted activity of a whole cell).

In total we obtain an estimate for the activity threshold due to ligand, receptor switching and methylation noise of $\Delta A / A^* = 0.06$. This is a lower bound for the activity threshold as other sources of noise, e.g. from stochastic phosphorylation and dephosphorylation events, are likely to further increase the threshold response. In the main text we used two different noise thresholds of 0.08 and 0.16, and show that Weber's law is valid for both.

Analysis of Weber's law and fold-change detection

To obtain an analytical formula for the threshold stimulus, we expand the receptor complex activity up to linear order about the steady state activity A^* [23]

$$\Delta A = \frac{\partial A}{\partial F}\bigg|_{F^*} \cdot \frac{\partial F}{\partial \ln c}\bigg|_{c_0} \cdot \frac{\Delta c_t}{c_0} = \alpha \cdot k \cdot \frac{\Delta c_t}{c_0}, \tag{8}$$

where F^* is the free-energy difference corresponding to A^*, $\alpha = \partial A/\partial F|_{F^*} = A^*(A^*-1)$ and

$$k = \frac{\partial F}{\partial \ln c}\Big|_{c_0} = \nu_a N \left[\frac{c_0}{c_0+K_a^{\text{off}}} - \frac{c_0}{c_0+K_a^{\text{on}}}\right] + \nu_s N \left[\frac{c_0}{c_0+K_s^{\text{off}}} - \frac{c_0}{c_0+K_s^{\text{on}}}\right], \tag{9}$$

which is constant $k \approx \nu_a N$ for $K_a^{\text{off}} \ll c \ll K_a^{\text{on}}$ and $k \approx \nu_s N$ for $K_s^{\text{off}} \ll c \ll K_s^{\text{on}}$ (and $k \approx 0$ otherwise). This results in Weber's law [24]

$$\Delta c_t = K \cdot c_0 \tag{10}$$

where $K = \Delta A/(\alpha k)$ is the constant Weber fraction in chemotaxis in the respective concentration regimes for threshold activity ΔA. Growing receptor-cluster sizes may explain that Weber's law extends to ~ 10 mM in regime I (Fig. 2E, see *Materials and Methods* and [25]) We also find that for background concentrations below K_a^{off}, Weber's law breaks down and the threshold stimulus approaches a constant,

$$\Delta c_t = \Delta A \cdot K_a^{\text{off}} K_a^{\text{on}} / [\nu_a N A^*(1-A^*)(K_a^{\text{off}} - K_a^{\text{on}})]. \tag{11}$$

Temporal exponential ramps

Main text Fig. 2C-F introduced the perception from the Weber-Fechner law with further details provided in Fig. S3A,B. This shows that exponential gradients are sensed best without sensory saturation. Here, we demonstrate that this can also be seen with exponential ramps (temporal gradients) in line with experiment.

Block *et al.* showed that temporal exponential ramps, i.e. exponentially increasing or decreasing concentrations of attractant in time, lead to an approximately constant (time-independent) rotational bias of the motor [26]. Hence, the time-dependent concentration input serves to probe the interplay of signaling and adaptation dynamics in the chemotaxis pathway. Here, we look at exponential ramps $c(t) = c_0 e^{\pm rt}$, with the initial concentration c_0 and rates $+r$ and $-r$ for up and down ramps, respectively. This time-dependent stimulus is used as input for the dynamic MWC model for mixed receptor complexes, whose activity $A(c, m)$ depends on the time-dependent concentration $c(t)$ and average receptor methylation level $m(t)$ (see main text). Fig. S3C shows simulated time courses; for slow ramp rates the adaptation dynamics catches up with the changing concentration, resulting in a new steady-state of the activity characterized by an approximately constant activity change. For high ramp rates, a plateau is reached for a limited period of time. Specifically, for down ramps the activity subsequently goes back to the adapted pre-stimulus value as the free-energy change due to the adaptation dynamics becomes faster than the free-energy change due to concentration changes. This is in in line with our expectation as the concentration approaches zero and further concentration changes decrease. For exponential up ramps, after an initial drop the activity increases when the concentration increases to values above the ligand dissociation constants of the Tar receptor (but below the ligand dissociation constants of the Tsr receptor). In this concentration regime, receptor complexes have low sensitivity towards MeAsp. Ultimately, at very high MeAsp concentrations, Tsr starts to bind and the activity decreases again.

Fig. S3D shows the plateau activity, i.e. the receptor complex activity for which dA/dt=0 is reached for the first time, as a function of the exponential ramp rate. According to the dynamic MWC model,

there is no threshold rate below which the receptor complex activity remains at the pre-stimulus value, which had been suggested by early experiments [26].

To obtain additional insight, we analytically solved the dynamic MWC model for small deviations from the adapted activity A^*, e.g. valid for small ramp rates. For simplicity, we restrict the concentration to $K_a^{\rm off} \ll c \ll K_a^{\rm on}$. In this regime, the free-energy difference Eq. 2 in the main text reduces to

$$F(m,c) = N \cdot \left[1 - 0.5\,m + \nu_a \ln\left(c/K_a^{\rm off}\right)\right]. \tag{12}$$

The rate of change of the free-energy difference is determined by (1) the rates of change of the receptor methylation level m and (2) changes of the concentration input c,

$$\frac{dF}{dt} = \frac{\partial F}{\partial m}\frac{dm}{dt} + \frac{\partial F}{\partial \ln c}\frac{d\ln c}{dt}. \tag{13}$$

The partial derivatives are given by

$$\frac{\partial F}{\partial m} = -\frac{N}{2} \tag{14}$$

$$\frac{\partial F}{\partial \ln c} = \frac{\nu_a N}{K_a^{\rm off}}. \tag{15}$$

This result can be approximated by the deviation of the rate of change of the methylation level from zero (i.e., the adapted steady-state) by expanding linearly about the steady state,

$$\frac{d(\delta m)}{dt} = \left(g_R + 3 g_B {A^*}^2\right)\delta A = \left(g_R + 3 g_B {A^*}^2\right)\frac{\partial A}{\partial F}\Delta F, \tag{16}$$

where $\Delta F = F - F^*$ is the deviation of the free-energy difference from its adapted value (corresponding to the adapted activity A^*). Furthermore, the derivative of the exponential concentration $c(t) = c_0 e^{\pm rt}$ is

$$\frac{d\ln c}{dt} = \pm r. \tag{17}$$

Subsequently, we obtain the following dynamics for the deviation of the free-energy difference from steady-state

$$\frac{d\Delta F}{dt} = -\frac{\Delta F}{\tau} \pm br, \tag{18}$$

where we introduced the parameters

$$\tau = \left[\frac{N}{2}A^*(1-A^*)\left(g_R + 3 g_B {A^*}^2\right)\right]^{-1} \tag{19}$$

$$b = \frac{\nu_a N}{K_a^{\rm off}} = \text{const.} \tag{20}$$

Equation 18 is solved by

$$\Delta F(t) = \pm\tau b r (1 - e^{\pm t/\tau}). \tag{21}$$

For times t larger than the time scale τ, i.e. after a transient period, this yields a time-independent change in the free-energy difference proportional to the ramp rate r

$$\Delta F(t) = \pm\tau b \cdot r \propto \pm r. \tag{22}$$

This result indicates that in the limit of small deviations from the adapted steady-state, the receptor complex activity assumes a new steady-state, which is shifted relative to its adapted pre-stimulus value when exposed to an exponential concentration ramp. Furthermore, the associated free-energy difference increases or decreases linearly with the rate r of the exponentially increasing or decreasing concentration, respectively. Consistent with our simulations shown in Fig. S3D, we do not obtain a threshold for the ramp rate, below which $F \approx 0$ (cf. *Inset*).

Extraction of physiological variance from FRET data

Principal component analysis (PCA) is a method to extract independent sources of variation. For FRET dose-response curves measured for Tar-only cells the largest contribution to the variation likely comes from the plasmid-induced protein expression as plasmid copy number can vary from cell to cell (here about 20-30) [27]. Using PCA, this type of physiologically irrelevant experimental variation can be removed from the variation in response amplitudes. In a D-dimensional space with the coordinates corresponding to the FRET activity at a particular concentration c_i ($i = 1, \ldots D$), each dose-response curve is represented by one D-dimensional data point. Plotting all dose-response curves results in a scatter plot [27]. PCA produces a new coordinate system centered around the mean value of the data, whose axes are orthogonal and a linear combination of the old axes. The coordinate axes are aligned along the directions of independent variation in the data. The coordinate transformation is obtained by diagonalizing the covariance matrix

$$C_{ij} = \frac{1}{M} \sum_{l=1}^{M} [A_l(c_i) - \bar{A}(c_i)][A_l(c_j) - \bar{A}(c_j)], \tag{23}$$

where c_i are ligand concentrations, A_l are the measured FRET activities and $\bar{A}(c_i)$ is the mean FRET activity measured at concentration c_i. The covariance matrix is diagonalized by $V^{-1}CV = U$, where V is the matrix containing the eigenvectors of C and U is a diagonal matrix of eigenvalues λ_i ($i = 1, \ldots, D$). The eigenvectors are the principle components of the data. Typically, most of the variation in the data is described by a small number of principle components, such that PCA can be used for a dimensional reduction of the data. Importantly, in our case the first principal component describes overall amplitude variation of the dose-response curves, likely due to variable gene expression from a plasmid.

Parameters of the fit of Eq. 11 in the main text to the variance in the FRET data without λ_1 are listed in Supporting Table S1 with confidence intervals and χ^2 quality-of-fit test). This table also contains parameters when fitted to the total variance (including λ_1).

Parameter fitting with assessment of confidence

Fits of the model in Eq. 11 to the variance in FRET activity, either including or excluding the first principal component of the noise (likely representing gene-expression noise [27]), were conducted in logarithmic parameter space. Parameter values α_1, α_2, and α_3 with confidence intervals and χ^2-quality-of-fit test are given in Table S1. Parameters were estimated by a Levenberg-Marquardt least-squares minimization using the Matlab function *nlinfit*.

Note that in Table S1 for WT 2 (0 mM) and QEEE we did not fit a separate switching noise term as the shapes of switching and internal noise are similar for these dose-response curves (indicated by NA). However, both are internal noise sources and need not be distinguished in Eq. 9-10. Ligand noise was not evaluated for WT (0 mM) due to the pre-factor c_0 in the ligand variance yielding zero input noise. In addition, we used the χ^2 quality-of-fit test to assess the quality of fit of Table S1. The χ^2 statistic is defined by the difference between model and data as $\chi^2 = 1/\sigma^2 \sum_i (m_i - e_i)^2$, where i denotes input concentration c_i, m_i is the predicted variance in FRET according to Eq. 11 and e_i the experimentally measured variance in FRET (cf. Fig. 3). The variance in FRET variance σ^2 was estimated from all residuals $m_i - e_i$. We calculated 95% confidence intervals using the profile-likelihood method [28].

For fitting the predicted input distributions in Fig. 3 and Fig. S4, we fitted in linear parameter space using a Levenberg-Marquardt least-squares estimation (Matlab function *nlinfit*). For each parameter we calculated a 95% confidence interval using the *nlparci* function in Matlab. Parameter values and confidence intervals are given in Table S2. The robustness of our predicted broad distribution of exponential gradients was tested in Fig. S10.

Fit of log-normal distributions to predicted input distributions

The predicted input distributions for higher receptor-modification states, i.e. QEQE, QEQQ, QQQQ and WT 2 adapted to 0.1 mM, can be fit by log-normal distributions

$$p_{\ln}(c;\mu,\sigma^2) = \frac{1}{c\sqrt{2\pi\sigma^2}} \exp\left(-\frac{[\ln(c)-\mu]^2}{2\sigma^2}\right). \tag{24}$$

The log-normal distribution is characterized by two parameters, μ and σ^2. The mean of a log-normally distributed concentration is $\langle c\rangle = e^{\mu+\sigma^2/2}$ and its variance is $\langle c^2 - \langle c\rangle^2\rangle = (e^{\sigma^2}-1)e^{2\mu+\sigma^2}$. Hence, the ratio of variance and squared mean can be expressed in terms of only one parameter, σ^2, producing $\langle c^2 - \langle c\rangle^2\rangle/\langle c\rangle^2 = e^{\sigma^2}-1$. Parameters of the log-normal fit of predicted input distributions of concentrations in Fig. 3C are listed in Table S2 with confidence intervals. As can be seen from the table, the parameter σ^2 is roughly constant for all strains, leading to the scaling behavior of the input distributions discussed in the main text. This table also contains the parameters of the log-normal fits when the first principal component (λ_1) is included, as well as for the Laughlin model (no input noise, but constant, uniform output noise; Figs. S4 and S10). All these fits show similar results, demonstrating robustness of our predictions from information theory.

Predicted distributions of inputs and outputs in the presence of uniform output noise

In order to better understand the role of the noise in the dose-response curve we compare our predicted distributions of inputs and outputs (in Fig. 3 in main text) with the theoretical results from zero input and uniform output noise (Fig. S1B) derived by Laughlin [10] (Fig. S4). Note that the magnitude of the uniform output noise does not affect the results, and hence is arbitrary. In the Laughlin model the output distributions are constant (Fig. S1B), while our model with variable noises sometimes shows bimodal output distributions (Fig. 3 in main text). The latter demonstrates that the sensory system avoids outputs corresponding to regions of the dose-response curve with large noise. Despite these differences in the distributions of outputs, both models produce similar distributions of inputs, with the distributions from the Laughlin model slightly narrower. This shows, as expected, that input noise and variable output noise lead to a broadening of the predicted distributions. Fig. S4 shows that the scaling of input distributions for the various receptor-modification levels also works for the Laughlin model.

Chemotactic index and relation to drift

The chemotactic index (CI), averaged over a short trajectory, is defined as the ratio between the distance traveled by a cell in the direction of the gradient and the total distance traveled,

$$\text{CI} = \frac{\sum_k l_k \cos\theta_k}{\sum_k l_k}, \tag{25}$$

where l_k is the run length and θ_k is the angle between traveled and gradient direction during run k (see Fig. S5A,B). CI thus encodes information on the trajectories but not on time required by cell to move up the gradient. For this reason the final biological output is usually considered to be the drift velocity which is defined as the average velocity swimming up the gradient [29]

$$v_d = \frac{\sum_k l_k \cos\theta_k}{\sum_k (t_k^{\text{run}} + t_k^{\text{tum}})}, \tag{26}$$

where t_k^{tum} and t_k^{run} are the tumbling and running times, respectively. However, CI is directly linked to the drift velocity v_d via

$$\text{CI} = \left(1 + \frac{\tau^T_{tum}}{\tau^T_{run}}\right)\frac{v_d}{v_{run}} \tag{27}$$

where $\tau^T_{tum} = \sum_k t_k^{\text{tum}}$ and $\tau^T_{run} = \sum_k t_k^{\text{run}}$ are the total times of tumbling and running, respectively, and $v_{run} \approx 20\mu\text{m}\ s^{-1}$ is the running velocity, assumed to be constant.

Comparison between simulations of swimming bacteria and experiments

To demonstrate the reliability of RapidCell software [22] we compare experiments with simulations of swimming bacteria. In particular, we first compared the CI between experiment and simulation in the same gradients and the drift velocity of the cells up the gradients (Fig. S5C,D). The experimental data was obtained from bacteria swimming in linear gradients in a microfluidic device [30].

Furthermore, we revaluated the distribution of clockwise (CW) and counter-clockwise (CCW) flagella rotation intervals for adapted cells. Fig. S5E,F show that our distributions match well the results from Block *et. al.* [26]. These tests, in additions to comparisons in [22], indicate that our simulations describe realistic swimming of bacteria.

Calculations of chemotactic index and drift velocity for different linear gradients

We studied the CI in linear gradients using RapidCell software [22] in the rectangular box described in *Materials and Methods*. Specifically, to calculate the CI we used schemes in Fig. S5A,B. For the case without rotational diffusion, the run length l_k is well defined (Fig. S5A). However, when simulated with rotational diffusion, as done in main text Figs. 4 and 5, we use a linear-piecewise trajectory with time step $\Delta t = 1$s to compute the CI (Fig. S5B). We then average the CI over many trajectories for a given average ligand concentration.

We make the following observations. First, we note that simulations without rotational diffusion (Fig. S6A) have a significantly larger CI than simulations with rotational diffusion (Fig. S6), suggesting once more that there is a well-defined maximum of CI. This maximum is broad for steep gradients and more well-defined for shallow gradients. Hence, we identified three different regimes in linear gradients. At low concentrations (to the left of maximum) the relative gradient is high but the CI can be relatively small, which is due to saturation of the receptor signal. This leads to long runs which may not always be well aligned with the gradient. In contrast, at high concentrations the relative gradient is low and hence cells are almost perfectly adapted, leading to a small CI as the gradient is invisible to the cells. This regime has additional features above ~ 0.5 mM, which coincides with the K_a^{on} value of the Tar receptors. At this concentration receptors become saturated and do not respond to attractant anymore. Furthermore, around 0.6 mM receptors become fully methylated and cannot adapt anymore. Hence, above 0.6 mM cells with fully methylated receptors only run. Both features lead to low CI values, which, however, do not drop to zero for very steep relative gradients. We interpret this observation as follows: Theoretically we expect for the concentration region around 0.6 mM that each run up the gradient leads to receptor saturation and, that each run down the gradient quickly leads to a tumble and reorientation. As a result, all cells in this region will eventually pick the right direction. We can imagine this to work as a reflecting mirror: all cells going in the wrong direction (down the gradient) will come back (and then swim up the

gradient) but not vice versa. The limiting value of CI is estimated to be

$$\langle \text{CI} \rangle = \frac{1}{2\pi} \int_{-\frac{\pi}{2}}^{\frac{\pi}{2}} \cos\theta \, d\theta = \frac{2}{\pi} \simeq 0.6, \tag{28}$$

which is close to the values in Fig. S6A for large relative gradients without rotational diffusion. The value is limited to around 0.6 since the alignment of the runs with the gradient is very inaccurate as all cells roughly swimming up the gradient keep running. The limiting value gets even smaller with rotational diffusion since cells accidentally going accurately up the gradient loose their sense of direction.

Finally, Fig. S6C shows the comparison of the CI and drift velocity v_d for trajectories with specific modification levels in different linear gradients, suggesting very similar trends in the two quantities.

Comparison of sampled input distribution with prediction

In this section we report further results about the sampled input concentrations of swimming bacteria from simulations in linear gradients in comparison with the predicted distributions from information theory (cf. main text Fig. 5A). Specifically, we calculated their overlap, the CI and the drift velocity for different relative gradients. Note that Tar-only cells have an adapted activity value $A^* \simeq 0.3$ which is smaller than activity 0.5 at the steepest region of the dose-response curve. As a result the predicted c^* values from information theory (mode of log-normal distribution) are below the mode of the sampled input distributions from simulations. Hence, to allow comparison of predictions and simulations, we shifted the predicted distribution to match the modes. Although Tar-only cells are genetically engineered, even wild-type cells have A^* below 0.5 ($\simeq 0.4$ [25]). This reduction relative to the optimal value from information theory allows smaller tumbling time without net movement [25].

To understand the origin of Fig. 5B in the main text, we present additional simulation results for modification levels $m^* = 4$ (QEQE) in Fig. S7, $m^* = 4.6$ (WT 2 in 0.1 mM) in Fig. S8, and $m^* = 6$ (QEQQ) in Fig. S9. However, we do not show results for $m^* = 8$ (QQQQ) since the corresponding adapted concentration $c^* \simeq 600\mu$M is higher than $\sim K_a^{\text{on}} \simeq 500\mu$M, leading to unrealistic long runs due to saturation. However, wild-type cells with multiple receptor types chemotax in concentrations higher than 500μM.

References

1. Olshausen BA, Field DJ (2000) Vision and the coding of natural images. Am Sci 88: 238-245.
2. Dunn FA, Rieke F (2006) The impact of photoreceptor noise on retinal gain controls. Curr Opin Neurobiol 16: 363-370.
3. Laughlin SB (1987) Form and function in retinal processing. Trends Neurosci 10: 478-483.
4. Laughlin SB (1989) The role of sensory adaptation in the retina. J Exp Biol 146: 39-62.
5. Johnson JK, Hsiao SS, Yoshioka T (2002) Neural coding and the basic law of psychophysics. Neuroscientist 8: 111-121.
6. Dehaene S (2003) The neural basis of the Weber-Fechner law: a logarithmic mental number line. Trends Cogn Sci 7: 145-147.
7. Pugh EN, Nikonov S, Lamb TD (1999) Molecular mechanisms of vertebrate photoreceptor light adaptation. Curr Opin Neurobiol 9: 410-418.

8. Fein A, Payne R (1989). Phototransduction in limulus ventral photoreceptors: Roles of calcium and inositol trisphosphate.

9. Hayashi JH, Moore JW, Stuart AE (1985) Adaptation in the input-output relation of the synapse made by the barnacle's photoreceptor. J Physiol 368: 179-195.

10. Laughlin S (1981) A simple coding procedure enhances a neuron's information capacity. Z Naturforsch C 36: 910-912.

11. Detwiler PB, Ramanathan S, Sengupta A, Shraiman BI (2000) Engineering aspects of enzymatic signal transduction: photoreceptors in the retina. Biophys J 79: 2801-2817.

12. Tkacik G, Callan Jr CG, Bialek W (2008) Information flow and optimization in transcriptional regulation. Proc Natl Acad Sci U S A 105: 12265-12270.

13. Tkacik G, Callan Jr CG, Bialek W (2008) Information capacity of genetic regulatory elements. Phys Rev E 78: 011910.

14. Mehta P, Goyal S, Long T, Bassler BL, Wingreen NS (2009) Information processing and signal integration in bacterial quorum sensing. Mol Syst Biol 5: 325.

15. Ziv E, Nemenman I, Wiggins CH (2007) Optimal signal processing in small stochastic biochemical networks. PLoS ONE 2: e1077.

16. Tostevin F, ten Wolde PR (2009) Mutual information between input and output trajectories of biochemical networks. Phys Rev Lett 102: 218101.

17. Clausznitzer D, Endres RG (2011) Noise characteristics of the Escherichia coli rotary motor. BMC Syst Biol 5: 151-151.

18. Berg HC, Purcell EM (1977) Physics of chemoreception. Biophys J 20: 193-219.

19. Bialek W, Setayeshgar S (2005) Physical limits to biochemical signaling. Proc Natl Acad Sci U S A 102: 10040-10045.

20. Bialek W, Setayeshgar S (2008) Cooperativity, sensitivity, and noise in biochemical signaling. Phys Rev Lett 100: 258101.

21. Endres RG, Wingreen NS (2008) Accuracy of direct gradient sensing by single cells. Proc Natl Acad Sci U S A 105: 15749-15754.

22. Vladimirov N, Løvdok L, Lebiedz D, Sourjik V (2008) Dependence of bacterial chemotaxis on gradient shape and adaptation rate. PLoS Comput Biol 4: e1000242.

23. Endres RG, Wingreen NS (2006) Precise adaptation in bacterial chemotaxis through "assistance neighborhoods". Proc Natl Acad Sci U S A 103: 13040-13044.

24. Tu Y, Shimizu TS, Berg HC (2008) Modeling the chemotactic response of *Escherichia coli* to time-varying stimuli. Proc Natl Acad Sci U S A 105: 14855-14860.

25. Clausznitzer D, Oleksiuk O, Lovdok L, Sourjik V, Endres RG (2010) Chemotactic response and adaptation dynamics in Escherichia coli. PLoS Comput Biol 6: e1000784.

26. Block SM, Segall JE, Berg HC (1983) Adaptation kinetics in bacterial chemotaxis. J Bacteriol 154: 312-323.

27. Endres RG, Oleksiuk O, Hansen CH, Meir Y, Sourjik V, et al. (2008) Variable sizes of *Escherichia coli* chemoreceptor signaling teams. Mol Syst Biol 4: 211.

28. Raue A, Kreutz C, Maiwald T, Bachmann J, Schilling M, et al. (2009) Structural and practical identifiability analysis of partially observed dynamical models by exploiting the profile likelihood. Bioinformatics 25: 1923-1929.

29. Reneaux M, Gopalakrishnan M (2010) Theoretical results for chemotactic response and drift of E. coli in a weak attractant gradient. J Theor Biol 266: 99-106.

30. Masson JB, Voisinne G, Wong-Ng J, Celani A, Vergassola M (2012) Noninvasive inference of the molecular chemotactic response using bacterial trajectories. Proc Natl Acad Sci U S A 109: 1802-1807.
